# Microbial Fuel Cells for Direct Electrical Energy Recovery from Urban Wastewaters

**DOI:** 10.1155/2013/634738

**Published:** 2013-12-19

**Authors:** A. G. Capodaglio, D. Molognoni, E. Dallago, A. Liberale, R. Cella, P. Longoni, L. Pantaleoni

**Affiliations:** ^1^Dipartimento di Ingegneria Civile e Architettura (D.I.C.Ar.), Università degli Studi di Pavia, 27100 Pavia, Italy; ^2^Dipartimento di Ingegneria Industriale e dell'Informazione, Università degli Studi di Pavia, 27100 Pavia, Italy; ^3^Dipartimento di Biologia e Biotecnologie “L. Spallanzani,” Università degli Studi di Pavia, 27100 Pavia, Italy

## Abstract

Application of microbial fuel cells (MFCs) to wastewater treatment for direct recovery of electric energy appears to provide a potentially attractive alternative to traditional treatment processes, in an optic of costs reduction, and tapping of sustainable energy sources that characterizes current trends in technology. This work focuses on a laboratory-scale, air-cathode, and single-chamber MFC, with internal volume of 6.9 L, operating in batch mode. The MFC was fed with different types of substrates. This study evaluates the MFC behaviour, in terms of organic matter removal efficiency, which reached 86% (on average) with a hydraulic retention time of 150 hours. The MFC produced an average power density of 13.2 mW/m^3^, with a Coulombic efficiency ranging from 0.8 to 1.9%. The amount of data collected allowed an accurate analysis of the repeatability of MFC electrochemical behaviour, with regards to both COD removal kinetics and electric energy production.

## 1. Introduction

Fuel cells are devices that use a combustion reaction without resorting to a thermal process, thus achieving direct conversion of chemical energy (of a generic “fuel” or “substrate”) into electrical energy. In particular, microbial fuel cells (MFCs) directly convert the chemical energy, contained in an organic bioconvertible substrate, into electrical energy, through the mediation of exoelectrogenic bacteria that act as catalyser of the half-reaction of substrate oxidation [1, 2]. The first evidence of this phenomenon was discovered in 1911 by Potter [1], but very few practical advances were achieved in the field until the first patent of mediatorless MFCs, dated 1999 [3]. MFCs-like technology has already been used as an energy source in field applications such as environmental sensors or process biomonitoring [4–8]. Some applications to brewery wastewaters treatment are also reported [9, 10]. Application of MFCs to municipal wastewater treatment appears to provide a potentially attractive alternative to traditional treatment processes that may include indirect energy recovery from wastes (e.g., anaerobic digestion with methane fermentation), as these devices are suitable to operate with low concentration substrates and temperatures below 20°C [11]. Although MFCs were tested for the first time in 2004 by Liu et al. [12], as of today their main applications remain confined to laboratory-scale plants. The limiting factors for MFC application to natural scale plants are, in fact, high initial capital costs (especially for electrode construction and membranes) and the limited power density that can be achieved [13, 14].

MFC's working principle relies on splitting the semireactions of oxidation and reduction that make up a typical redox reaction, allowing them to occur in two different compartments. In the anodic compartment, exoelectrogen bacteria catalyse substrate oxidation and transfer the electrons, released from cellular respiratory chain, to a metal electrode (i.e., anode) [14]. Electrons then flow through an external electric circuit towards the cathodic compartment, where they reduce the terminal electron acceptor (TEA, usually oxygen) [15]. For each electron released at the anode, an H^+^ ion must reach the cathode through the electrolytic solution saturating the cell, in order to internally close the circuit and reestablish electric neutrality. Hence electrons and protons react with oxygen on the cathode, generating water vapour H_2_O [15].

The maximum current that can be produced by a MFC depends on the actual rate of substrate biodegradation, whereas maximum theoretical cell voltage (also called electromotive force or *emf*) depends on Gibbs free energy of the overall reaction and can be calculated as the difference between the standard reduction potentials of the cathodic oxidant (oxygen) and the chosen anodic substrate (e.g., as per reference [16]) [2, 14, 17]. However, the cell's *emf* is a thermodynamic value that does not take into account any internal losses [17]. Measured experimental values are always substantially lower than theoretical ones.

The typical MFC configuration is that of a dual-chamber cell, where the anodic and cathodic volumes (chambers) are separated by a protonic or cationic exchange membrane (PEM or CEM), which allows internal ionic fluxes but prevents mixing of anodic reducing solution and cathodic oxidant [17]. This membrane, however, is one of the principal cost factors in a MFC plant and increases the cell's internal resistance (i.e., the measurement of all internal voltage losses, occurring when current flows throughout the system) [13, 14, 17]. For this reason, current research on MFCs has shifted towards the use of single-chamber, membraneless cells (so called SC-ML-MFCs), where the cathode is directly exposed to the atmosphere (so called air-cathode) [13]. Dual-Chamber MFCs are usually still investigated when the specific aim is to exploit the cathodic reduction semireaction, for the removal of nutrients from wastewater or groundwater lacking organics (e.g., [18–20]).

In the case of SC-MFCs, the cathode has proved to be the most critical component of the process. The cathode must in fact provide the interface between three separate phases: the oxidant gas (atmospheric oxygen), the liquid electrolyte (containing the moving H^+^ ions), and the solid conductor (external circuit), through which electrons flow. It is therefore likely to be the cathode limiting electrode for power generation [13]; several studies looked at ways to improve its electrical performances, avoiding at the same time the adoption of expensive chemical catalysts and/or ionic exchange membranes/resins [21–25]. Determination of the optimal material for electrode construction and definition of the most suitable dimensional ratios between electrode surfaces and cell volume are still object of investigation [13].

### 1.1. MFC Reactor Parameters

In order to compare the performance of different reactor configurations and electrodes, a series of parameters and experimental methods have been proposed, to determine the bioelectrochemical performances of MFCs. One of the more encompassing reviews on this subject has been written by Logan et al. [17, 26]. Firstly, the mean power (and current) produced by the cell must be normalized by a relevant geometric characteristic of the reactor that could be one of the electrodes' surface area or the volume of anodic chamber (when dealing with SC-MFCs). In this study we have chosen to express current density with respect to cathode surface (the limiting electrode in our process) and power density with respect to the total reactor volume.

The polarization curve is a synthetic method to analyse the behaviour of a MFC [17]: the curve represents the dependence of cell's voltage on the electrical current flowing in the circuit and allows to estimate the values of electrode overpotentials and the internal resistance of the cell, representing an overall measurement of cell's internal voltage losses and defined, geometrically, by the slope of the linear region of the polarization curve [26]. The power curve is calculated from the polarization curve and describes the power output of the cell as a function of the current. Usually it has a parabolic shape with a single point of maximum (called Maximum Power Point or MPP), which occurs when the external resistance of the circuit equals internal resistance of the cell.

From the wastewater treatment engineer's point of view, it is possible to evaluate the substrate conversion rate of a MFC, in terms of chemical oxygen demand (COD), through the determination of the COD removal efficiency or, better, of its removal rate (thus taking into account the retention time of the substrate in the cell).

Finally, an important parameter for the evaluation of MFCs performance is its Coulombic efficiency, defined as the ratio of actual transferred electric charge and its maximum value obtainable, if all of the substrate's removal were to produce a current [17].

In this study, a laboratory-scale SC-MFC, with an air-cathode of novel design, operating in batch mode with internal recirculation was built and operated. The cell was sequentially fed with different wastewaters, both synthetic and natural, in order to test exoelectrogenic bacteria behaviour under various substrate load conditions. The aims of the study conducted were primarily to characterize the cell under both an electrical and a substrate removal point of view, through the construction of polarization and power curves, determination of COD removal rates, mean electrical power, and Coulombic efficiencies.

## 2. Materials and Methods

### 2.1. MFC Construction and Operation

**Figure 1:**
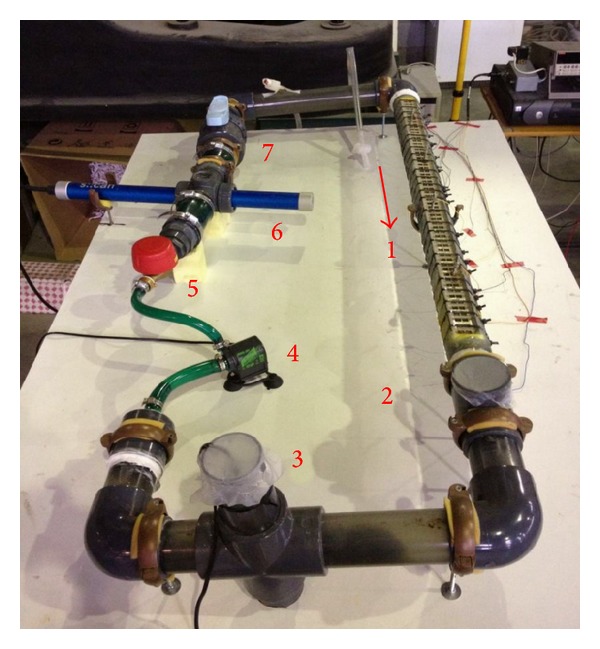
MFC prototype used in laboratory: (1) active section of the plant, where electrodes are situated; (2) sampling port; (3) discharge tap, equipped with thermostat; (4) recycle pump; (5) volumetric flow meter; (6) spectrophotometric probe; (7) ball valve for flux tuning.

The prototype utilized in the experimentation was an in-house built, membraneless SC-MFC, with an air cathode, operating in batch mode with internal recirculation due to the unit volume (Figure 1). The configuration adopted is different from those SC-MFC typically studied in the literature, which are usually of much smaller capacity (e.g., [12, 21, 22, 25]), as this cell has a built-in hydraulic circuit in which the waste is kept moving by means of a low-flow pump. This was chosen, in order to eventually shift from batch mode, in which tests were carried out so far, to continuous operation mode, once the overall behaviour of the process became more clear. The internal volume of the cell was 6.9 L.

**Figure 2:**
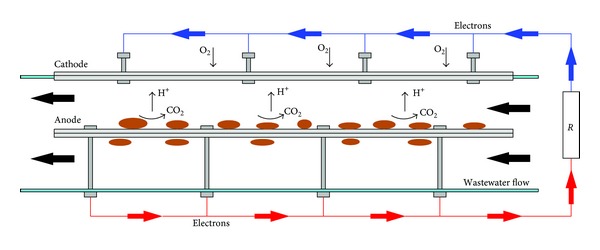
Working principle of MFC prototype.

The anode consists of carbon cloth (nonwet proofed, E-Tek), wrapped around a stainless steel bar (geometric area: 230 cm^2^), positioned inside the pipe. It is kept in position by a series of threaded rods, which allow also to modify the electrodes' distance. The cathode was made of carbon cloth (wet proofed with 30% wt PTFE, E-Tek), inserted between two stainless steel frames (area exposed to atmosphere: 76 cm^2^), positioned at the upper side of the (cut) pipe composing the circuit. This way, it represents a solid interface between the reducing wastewater, filling the cell, and the oxidant atmosphere. Electrodes at 3 cm distance were electrically connected by an external circuit, while the internal electrolyte function was carried out by the free volume of the separating liquid (Figure 2).

The cathode was kept wet at all times and as horizontal as possible, in order to not partialize its electroactive section. The system's tubing was made of clear Plexiglas (internal diameter: 59 mm), in order to visually control the process. The MFC was normally covered with an opaque cloth, to prevent the growth of microalgae on internal surfaces (that was observed initially), and all openings were sealed with Parafilm, to avoid oxygen diffusion inside the cell. Wastewater was kept moving at a flow-rate of 3.5 L/min, so the mean flow velocity between electrodes was about 2 cm/s, in order not to shear away exoelectrogen bacteria colonizing the submerged anode.

### 2.2. Inoculum Procedure and Wastewater Composition

The cell was initially inoculated with 0.5 L of mixed sludge (aerobic from aerated basin and anaerobic from the digester) and filled with urban wastewater, both spilled from the local municipal wastewater treatment plant (WWTP). Several authors agree in fact that the inoculation with mixed biomass seed assures a better efficiency of the MFC (both in electric and COD removal terms) when complex organic substrates are used as fuel [15, 27–30]. Initially the cell was fed with a simple synthetic wastewater (hereafter SW1), containing CH_3_COONa as carbon source, (NH_4_)_2_SO_4_ and Na_2_HPO_4_ as macronutrients, with COD : N : P ratio equal to 100 : 12 : 1.6 (typical of municipal wastewater), and an organic load to the cell equal to 0.5 kg_COD_/kg_VSS_/d (where VSS means volatile suspended solids). Acetate is largely applied in MFC experimental studies, since it is easily oxidised by the exoelectrogen anodic biomass and it is inert towards alternative microbial conversions [31]. This phase lasted for about two months. After this observation period, the feed was switched to a more complex synthetic waste (hereafter SW2), prepared in order to mimic presettled domestic wastewater (a slightly modified version of *Syntho* [32]). Concentrations and physicochemical characteristics of SW2 waste are shown in Table 1.

### 2.3. MFC Polarization Curves

Tracing the MFC's polarization and power curves was a key objective of this study, and several methods could be adopted for this purpose [17, 26]. In the course of our tests, it was decided to proceed in two ways: operating with a passive external load (i.e., variable resistor method) and with an active load (similar to a potentiostat scan) [33]. Although the latter provided interesting indication on the electrical behaviour of the MFC, most of the work herein described was done with the first method, as it was characterised by a simpler setup, and almost fully automated. Polarization curve measurements were conducted by means of a digitally controlled potentiostat, operating as a variable resistance in the range 30–1000 Ω. Voltage over each resistance was recorded using a multimeter until pseudo-steady state was reached (voltage variation less than 0.5 mV/min). The current output was then calculated by means of Ohm's law. The obtained polarization and power curves were used to determine the external resistance value from which maximum power output could be drained (i.e., internal resistance of the MFC).

### 2.4. MFC Operating Mode

Beyond the initial inoculum and bacterial growth period, the MFC was operated in batch mode with internal recirculation, with mean retention time (hereafter HRT) of the waste in the cell equal to 144 hours (6 days) for each treatment cycle (T.C.). Daily wastewater samples were taken from the anodic chamber and analysed for COD contents, while the same COD concentration and electric voltage across a fixed external resistance (*R*_ext_) were continuously monitored through dedicated online data acquisition systems. Liquid level within the cell was constantly monitored and if necessary adjusted with distilled water in order to assure closure of the internal electric circuit (i.e., cathode wetting). Polarization curves were recorded at the beginning of each cycle, according to the variable resistor method, the cell's internal resistance calculated, and *R*_ext_ adjusted manually to be as close as possible to that value. In this fashion, as much electrical energy as possible was gathered from the MF, as done in previous trials [34].

Slight changes to this standard protocol were adopted in some treatment cycles, in order to simplify operation schedule and/or monitor the behaviour of the cell under different conditions. Specifically

- the cell was not emptied completely at the end of the treatment cycle, but simply filled up with concentrated wastewater, to reach the desired COD concentration (in 2 instances, SW2 wastewater was adopted);
- polarization curves were obtained with the active load method (for information on the method refer to [33]);
- definition of a polarization curve every day, in order to follow changes of MFC internal resistance in time and to adjust accordingly *R*_ext_ (6 instances, all substrates tested). This was a first attempt of MPPT control implementation, which is currently still under study;
- measurement of only two wastewater samples for COD determination (at the beginning and end of the T.C.), when continuous COD recording from spectrophotometric probe (Spectro-lyser, from S-CAN Gmbh, Vienna, Austria) was available.

## 3. Parameters for Process Description

### 3.1. Wastewater Treatment Efficiency

Organic substrate removal rate, within any biological treatment system, is not constant but depends (among other parameters) on the organic loading of the reactor. The batch operation mode of the MFC did not allow to reach stationary conditions in the cell. To evaluate the experimental results, a first order degradation kinetics model, with expression

(1) $$\begin{array}{c} {\text{COD}}_{t}\;=\;a\cdot e^{-b\cdot t} \end{array}$$

was adopted, where COD_*t*_ (mg/L) represents the COD residual concentration in the cell after treatment time *t* (h), the parameter *a* (mg/L) is proportional to the influent COD concentration, *b* (h^−1^) is the kinetic constant of the biodegradation process. This was used to fit experimental data collected from both the laboratory chemical COD analysis and the spectrophotometric probe, which was previously calibrated to match them. For each treatment cycle the COD removal efficiency was calculated as

(2) $$\begin{array}{c} \eta =\frac{\left({\text{COD}}_{\text{in}}-{\text{COD}}_{\text{out}}\right)}{{\text{COD}}_{\text{in}}}\cdot 100, \end{array}$$

where COD_in_ (mg/L) is the influent concentration and COD_out_ (mg/L) is the concentration after treatment. As HRT was not always constant, a normalized COD removal rate

(3) $$\begin{array}{c} \text{CRR}=\frac{\left({\text{COD}}_{\text{in}}-{\text{COD}}_{\text{out}}\right)}{\text{HRT}} \end{array}$$

was calculated for comparison purposes. Simplified statistical analysis was finally performed, assuming a normal distribution hypothesis, in order to compare the biological behaviour of the MFC with respect to the different types of fed substrate.

### 3.2. Internal Resistance Calculation

Internal resistance of the MFC (hereafter *R*_int⁡_) was calculated with the power density peak method, described in Logan's [26], from measured power curves. Maximum power output, associated with that resistance, was then compared to the mean power actually measured during the wastewater treatment cycle.

### 3.3. Electric Energy Recovered and Coulombic Efficiency

For each batch cycle the temporal trend of MFC electrical behaviour was observed, relying on voltage measurement across the external resistive circuit and calculating current density (referred to cathodic surface) and power density (referred to reactor's total volume). Integrating measured electric power over batch treatment time, the total electric energy recovered for wastewater unit volume (E_recovered_, in kJ/m^3^) was determined.

Finally, in order to estimate the global efficiency of the bioelectrochemical process, the MFC's Coulombic efficiency, defined as the ratio between electron moles extracted as current and the total electron moles made available from substrate oxidation [26], was calculated. In case of a batch system, fed with a complex substrate, the calculation is based on wastewater COD concentration:

(4) $$\begin{array}{c} C_{E}=\frac{8\int I\cdot dt}{\left(F\cdot V_{\text{an}}\Delta \text{COD}\right)}\cdot 100, \end{array}$$

where *I* is the current (A), *F* represents Faraday's constant (96485 C/mol), and *V*_an_ is the cell internal volume (i.e., the volume of treated waste in L). ΔCOD is equal to the difference between COD_in_ and COD_out_ (values in g/L).

## 4. Results and Discussion

### 4.1. Testing with SW1 Wastewater

After the initial biomass growing period, the MFC was fully monitored for two weeks while feeding the anodic chamber with simple synthetic wastewater containing CH_3_COONa as the only oxidable compound (SW1). Theoretical *emf*, calculated assuming COD concentration of 300 mg/L, neutral pH, and temperature of 23°C (maintained constant by means of a thermostat), was equal to 1.1 V [26]. Experimental values of our MFC voltage at the external resistance of 1000 Ω (hereafter *E*_1000_) were always substantially lower (Table 2).

**Figure 3:**
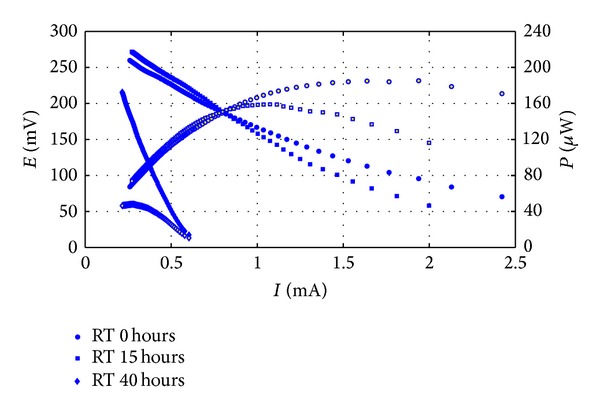
Electrodes polarization during treatment cycle 1. Filled points represent polarization curves, empty points the power curves.

Reasons for this probably consisted of high electrodes overpotentials (i.e., activation losses) and low ionic strength of the wastewater, indeed the linear region of polarization curve exhibited only a moderate gradient, which resulted in values of *R*_int⁡_ comprised between 49 and 630 Ω (Figure 3).

From Figure 3 it is clear that *R*_int⁡_ increased as the residence time of wastewater in the cell. That was mainly due to reduction of maximum extractable current, rather than Open Circuit Voltage variation. *E*_1000_ values remained quite constant around 220–270 mV, at least for the first 40 hours.  Table 3 shows final results of the continuous MFC monitoring, throughout the two batch treatment cycles performed with SW1.

**Figure 4:**
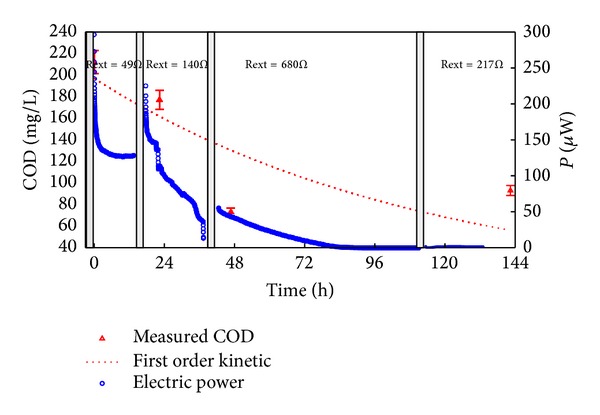
Continuous monitoring of treatment cycle 1. Grey bars are placed in correspondence of polarization curve measurements.

Both T.C.1 and -2 exhibited a rapid power drop after the first 72 hours of operation (Figure 4), when the energy recover had already reached 80% of its final value, though wastewater COD was still higher than 100 mg/L. T.C.2 showed a mean power output of 8.7 mW/m^3^, 58% higher than T.C.1, but this can be ascribed to a higher COD concentration in the influent, as both the kinetic constant and Coulomb efficiency determined for these cycles assumed almost the same values.

### 4.2. Testing with SW2 Wastewater

After the first two weeks, three treatment cycles with a more complex synthetic waste (SW2) were carried out. SW2 was designed to mimic behaviour of a natural substrate [32], but without the presence of toxic compounds and with limited COD oscillations. The cell was not completely emptied and cleaned at the end of each cycle but simply filled with anodic biomass and a concentrated dose of wastewater. This is in order to simulate a continuous operation of the cell, attempting to achieve maximum biomass growth at the anode and, eventually, maximum concentration of endogenous electron mediators in wastewater. Results are summarized in Table 4 (polarization curves) and Table 5 (continuous monitoring).

**Figure 5:**
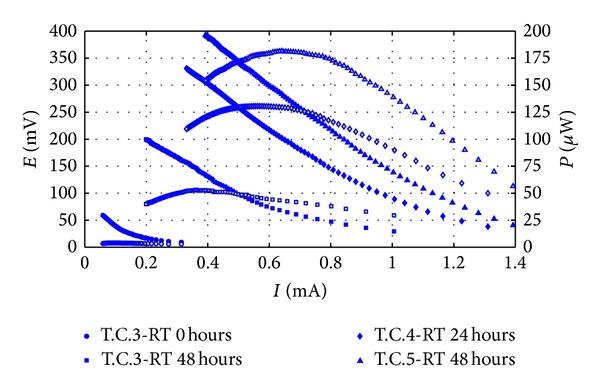
Anodic biomass growing throughout three consecutive treatment cycles with SW2, without emptying the cell. Filled points represent polarization curves, empty points the power curves.

Polarization curves showed how MFC electric behaviour improved by not emptying and cleaning the cell (Figure 5) but finally resulted in a *P*_max⁡_ of 25.9 mW/m^3^, almost the same value reached with SW1 under previous operating conditions.

Although from an analysis of the polarization curves it seems that the cell behaviour was not overly affected by the adopted feed waste composition, continuous monitoring revealed that both biological and electrochemical behaviour improved considerably utilizing SW2. The first order kinetic rate constant, *b*, and the COD removal efficiency reached very stable values (Table 5), while the average power production and Coulombic efficiency increased from one cycle to the next, proving how the MFC's biomass progressively improved its exoelectrogen characteristics.

## 5. Conclusions

A laboratory-scale SC-MFC, with air-cathode, operating in batch mode with internal recirculation, was built and studied. The MFC was fed with two different synthetic wastewaters, in order to test exoelectrogenic bacteria behaviour under various conditions. An average COD removal efficiency of 86% was achieved, with mean waste retention time (HRT) of 150 hours. The MFC produced an average power density of 13.2 mW/m^3^, with peaks of 20–30 mW/m^3^. Electrical energy recovered amounted to 7.9 kJ per m^3^ of treated waste. Coulombic efficiency was in the range 1-2%, with mean value of 1.2%. These values are lower than those achievable by chemical Fuel Cells; however, this is of relative importance since the intended fuel (urban wastewater) in this case is actually a waste that must be disposed of at a nonnegligible cost. The synthetic wastewater adopted (SW2), very similar to actual urban wastewater, showed good results in terms of experimental repeatability and will be useful for future investigations on MFC process and for benchmarking of the process when shifting to actual urban wastewater. Synthetic waste allowed also to test continuous flow operation showing an improvement of electric behaviour over time. This suggested that a well-designed continuous flow plant could ensure better bioelectrochemical performances than abatch one, once in steady-state conditions.

The results of this study will be applied to improve the design of the tested MFC and to switch to actual urban wastewater substrate operation. Although electric power generation was modest, this study shows that MFCs are feasible, although in need of improvement, process for urban wastewater treatment allowing direct energy recovery from a waste source.

## Figures and Tables

**Table 1:** SW2 synthetic wastewater composition. Compounds' mixtures were dry-stored in 50 mL sterile Falcon tubes until use, when they were dissolved into the proper amount of distilled water and then fed to the cell. Trace metals solution was added too (1 mL/L). Measured pH of the final solution was equal to 6.8 ± 0.6; conductivity was 1025.5 ± 128 *μ*S/cm.

Compounds	(mg/L)	COD (mg/L)	*N* (mg/L)	*P* (mg/L)
Urea	69.2	17.5	32.3	0.0
NH_4_Cl	9.6	0.0	2.7	0.0
CH_3_COONa	59.8	47.3	0.0	0.0
Peptone	13.1	15.7	0.5	0.0
KH_2_PO_4_	17.6	0.0	0.0	2.4
FeSO_4_·7H_2_O	4.4	0.0	0.0	0.0
Sucrose	92.0	103.3	0.0	0.0
Milk powder	87.6	106.8	5.2	0.9
Yeast	39.4	39.4	4.7	0.0
MnSO_4_·H_2_O	0.1	0.0	0.0	0.0
NiSO_4_·6H_2_O	0.3	0.0	0.0	0.0
ZnCl_2_	0.2	0.0	0.0	0.0

Total	—	330.0	45.4	3.2

**Table 2:** MFC feeding with SW1 wastewater. Polarization curves overview of treatment cycle 1. *E*_1000_ represents the cell voltage at external resistance of 1000 Ω, *I*_30_ represents the current intensity at external resistance of 30 Ω (i.e., potentiometer range).

T.C.	HRT (h)	*E*_1000_ (mV)	*I*_30_ (mA)	*I*_30_ (mA/m^2^)	*P*_max⁡_ (*µ*W)	*P*_max⁡_ (mW/m^3^)	R_int⁡_ (Ω)
1	0	260	2.4	317.9	185.2	26.5	49.3
1	15	272	2.0	264.9	159.0	22.7	137.8
1	40	216	0.6	79.5	48.2	6.9	630.4
1	112	32	0.2	26.5	1.1	0.2	226.5

**Table 3:** MFC feeding with SW1 wastewater. Continuous monitoring results and bioelectrochemical performances calculation.

T.C.	COD_in_ (mg/L)	HRT (h)	*R*_ext_ (Ω)		*b* (h^−1^)	*η*_ COD_ (%)	CRR (mgL^−1^d^−1^)	*P*_mean_ (mW/m^3^)	*E*_recovered_ (kJ/m^3^)	*C*_*E*_ (%)
1	212	142	variable	49–680	8.82*E* − 03	56%	20.1	5.5	2.8	1.8%
2	309	162	fixed	148	8.31*E* − 03	74%	33.9	8.7	5.0	1.7%

Average	261	152	—	—	8.57E − 03	65%	27.0	7.1	3.9	1.8%
st. dev.	69	14	—	—	3.61E − 04	13%	9.8	2.3	1.5	0.0%

**Table 4:** MFC feeding with SW2 wastewater. Polarization curves overview throughout three consecutive treatment cycles. *E*_1000_ represents the cell voltage at external resistance of 1000 Ω, *I*_30_ represents the current intensity at external resistance of 30 Ω.

T.C.	RT (h)	*E*_1000_ (mV)	*I*_30_ (mA)	*I*_30_ (mA/m^2^)	*P*_max⁡_ (*µ*W)	*P*_max⁡_ (mW/m^3^)	*R*_int⁡_ (Ω)
3	0	60	0.3	39.7	4.0	0.6	452.5
3	48	200	1.0	132.5	53.1	7.6	403.4
4	24	331	1.3	172.2	130.6	18.7	413.3
5	48	400	1.4	185.4	181.6	25.9	442.7

**Table 5:** MFC feeding with SW2 wastewater. Continuous monitoring results and bioelectrochemical performances calculation.

T.C.	COD_in_ (mg/L)	HRT (h)	*R*_ext_ (Ω)		*b* (h^−1^)	*η*_ COD_ (%)	CRR (mgL^−1^d^−1^)	*P*_mean_ (mW/m^3^)	*E*_recovered_ (kJ/m^3^)	*C*_*E*_ (%)
3	331	143	fixed	470	1.36*E* − 02	86%	47.7	8.2	4.9	0.8%
4	330	165	fixed	470	1.20*E* − 02	86%	41.4	11.6	6.9	1.1%
5	330	143	fixed	470	1.41*E* − 02	87%	48.0	19.7	11.8	2.0%

Average	330	150	—	—	1.32E − 02	86%	45.7	13.2	7.9	1.2%
st. dev.	1	13	—	—	1.10E − 03	0.5%	3.7	5.9	3.6	0.6%
